# Comparative Investigation of Thromboelastometry and Thrombin Generation for Patients Receiving Direct Oral Anticoagulants or Vitamin K Antagonists

**DOI:** 10.3390/diagnostics14222553

**Published:** 2024-11-14

**Authors:** Armando Tripodi, Marco Capecchi, Erica Scalambrino, Marigrazia Clerici, Barbara Scimeca, Pasquale Agosti, Paolo Bucciarelli, Andrea Artoni, Flora Peyvandi

**Affiliations:** 1 Fondazione IRCCS Ca’ Granda Ospedale Maggiore Policlinico, Angelo Bianchi Bonomi Hemophilia and Thrombosis Center, 20122 Milano, Italy; m.capecchi87@gmail.com (M.C.); erica-scalambrino@policlinico.mi.it (E.S.); marigrazia.clerici@policlinico.mi.it (M.C.); barbara.scimeca@cardiologicomonzino.it (B.S.); pasquale.agosti@unimi.it (P.A.); paolo.bucciarelli@policlinico.mi.it (P.B.); andrea.artoni@policlinico.mi.it (A.A.); flora.peyvandi@unimi.it (F.P.); 2 Fondazione Luigi Villa, 20122 Milano, Italy; 3 Department of Pathophysiology and Transplantation, Università degli Studi di Milano, 20122 Milano, Italy

**Keywords:** acquired coagulation disorders, coagulation inhibitors, deep vein thrombosis, thrombin, thrombosis

## Abstract

**Background**. Alterations induced by direct oral anticoagulants (DOACs) or vitamin K antagonists (VKAs) to thromboelastometry and thrombin generation are not well defined. We performed a simultaneous investigation of thromboelastometry and thrombin generation for patients who were chronically anticoagulated with DOACs or VKAs. **Methods**. A total of 131 patients on DOACs [apixaban (*n* = 37), rivaroxaban (*n* = 34), dabigatran (*n* = 30), edoxaban (*n* = 30)] and 33 on VKAs were analyzed. Whole blood was analyzed for thromboelastometry and plasma was analyzed for thrombin generation. **Results**. While the thromboelastometry clotting time (CT) was responsive to the hypocoagulability induced by DOACs or VKAs, clot formation time and maximal clot formation were not. Cumulatively, the parameters denoting the velocity of thrombin generation (lag time, time-to-peak) were relatively less responsive to the hypocoagulability induced by VKAs than DOACs. Conversely, the parameters denoting the quantity of thrombin generation [peak-thrombin and the endogenous thrombin potential (ETP)] were more responsive to the hypocoagulability induced by VKAs than DOACs. Apixaban showed relatively small differences (peak vs. trough) in the plasma concentration and a relatively small (peak vs. trough) difference of hypocoagulability when assessed by the CT or the ETP. The CT and the ETP were strongly correlated with DOAC concentrations or with the VKA-INR. **Conclusions**. DOACs and VKAs altered thromboelastometry and thrombin generation to an extent that probably reflects the mode of action of these drugs and may also have practical implications for patients’ management. Apixaban showed a small difference of hypocoagulability (peak vs. trough), suggesting a more stable anticoagulation over the daily course of treatment. Based on the correlations of the CT or the ETP vs. the DOAC concentrations, we estimated that critical values of the CT or the ETP would correspond to DOAC concentrations of 400 or 20 ng/mL. Whenever dedicated tests for DOAC concentrations are not available, the CT or the ETP can be used as surrogates to evaluate the level of anticoagulation induced by DOACs.

## 1. Introduction

Direct oral anticoagulants (DOACs) were introduced many years ago for the treatment/prevention of thrombosis in cardiovascular diseases. Randomized controlled trials involving thousands of patients showed that DOACs are at least as effective as VKAs for VTE treatment/prevention or for the prevention of ischemic stroke and systemic embolism in patients with non-valvular atrial fibrillation (AF). Furthermore, DOACs are associated with a lower risk of life-threatening bleeding, such as intracranial bleeding [1,2]. Because of the above favorable characteristics, DOACs are replacing VKAs for most indications [3]. Although DOACs and VKAs affect, to some extent, hemostasis tests [4], no direct comparison on the effect that different anticoagulants may have on global coagulation procedures has been reported. Furthermore, little is known of the effect that DOACs may have on global procedures at peak and trough plasma concentrations. The primary aim of this study was to evaluate the distribution of the main parameters of thromboelastometry and thrombin generation assays (TGA) between patients on VKAs and DOACs and within DOACs. The secondary aim of this study was to look at correlations between the parameters of thromboelastometry or TGA and the plasma concentrations of DOACs. Scrutiny of the results may provide important clues to understand the mechanisms of action of these anticoagulants. The results can also provide information that may be used for patients’ management.

## 2. Patients

The protocol of this cross-sectional study was approved by the local ethics committee. Blood was drawn on the occasion of clinical visits and the patients gave their informed consent to be included in the study. We included patients attending the outpatient clinic for the control of anticoagulation of the IRCCS Ca’ Granda Maggiore Hospital Foundation, Milano, Italy. The patients were on anticoagulants for the treatment/prevention of VTE or for the prevention of stroke and systemic embolism in AF and were on chronic anticoagulation at the time of the blood collection (at least three months from the initiation of VKAs and two months for DOACs). Patients on DOACs were invited to come to the outpatient clinic just before the administration of the daily dose. The blood samples were collected and the patients were instructed to take the next dose immediately and to wait for two hours, after which, a second blood drawing was performed. The first blood draw represented the trough level, and the second represented the peak. The blood was collected in vacuum tubes (Vacuette, Greiner Bio-One International, Kremsmünster, Austria) containing a 1/10 volume of trisodium citrate 109 mM. A portion of whole blood was taken for the measurement of thromboelastometry and was stored at room temperature until testing, which was performed no later than one hour from blood collection. The rest was centrifuged for 20 min at 3000× *g* (controlled room temperature). The plasma was aliquoted in plastic capped tubes, immersed in liquid nitrogen, and stored at −70 °C until testing for the international normalized ratio (INR) for VKAs, DOAC concentrations, and thrombin generation.

## 3. Methods

To limit the effect of methodological variability, the laboratory testing for the frozen plasma was performed in batch, including, at each test occasion, equal numbers of samples from each category of the investigated patients.

### 3.1. Thromboelastometry

The whole blood was subjected to the measurement of thromboelastometry parameters using rotation thromboelastometry (RoTem, Werfen, MA, USA) with the reagents and instructions provided by the manufacturer. We evaluated the following parameters. (i) Clotting time (CT), representing the time (seconds) needed for the blood to start clotting. (ii) Clot formation time (CFT), representing the time (seconds) needed for the clot to reach a defined amplitude and being essentially concerned with the velocity of the clot formation. (iii) Maximal clot firmness (MCF), representing the maximal firmness and strength of the clot (mm). The above parameters were recorded using reagents to explore the extrinsic (EXTEM) or intrinsic (INTEM) pathways of coagulation.

### 3.2. Thrombin Generation

TGA was performed according to Hemker et al. [5], using a homemade method [6] that consists of plasma activated by small amounts of tissue factor (1 pM), derived from Recombiplastin 2G (Werfen, MA, USA), synthetic phospholipids (1 µM) (AvantiPolar, Alabaster, AL, USA), and calcium chloride. Thrombin generation was continuously monitored by a fluorogenic substrate (417 µM) (Backem, Switzerland) by means of a dedicated fluorimeter (Fluoroskan Ascent, ThermoLab Systems, Helsinki, Finland) and dedicated software (Thrombinoscope, Thrombinoscope, the Netherlands) which converts the instrument signals into thrombin concentrations and constructs a thrombin generation curve. We recorded the following parameters. (i) Lag time, representing the time (minutes) needed for the first trace of thrombin to appear upon activation. (ii) Time-to-peak, representing the time (minutes) needed for thrombin concentration to reach the peak. (iii) Peak-thrombin, representing the highest concentration of thrombin (nM thrombin). (iv) The area under the curve (nM thrombin × minutes), also called the endogenous thrombin potential (the ETP), representing the net amount of thrombin that plasma can generate under the driving forces of the procoagulants, as opposed by anticoagulants.

### 3.3. The Measurement of the Levels of Anticoagulation

The INR for patients on VKAs was measured using Recombiplastin 2G on ACLTop (Werfen), and the DOAC concentrations were measured using dedicated assays that included dilute thrombin, time for dabigatran, and anti-FXa assays for rivaroxaban, apixaban, and edoxaban (Werfen). The results of the DOAC concentrations were expressed as the drug concentration equivalent (ng/mL) using calibration curves that were constructed by testing the dilutions of certified standards that were specific for each drug.

## 4. Statistical Analyses

The data are reported as medians and interquartile ranges (IQRs). The percentage of the DOAC concentration measured at trough relative to that measured at peak that was obtained for each patient was calculated by dividing the trough concentration by the peak concentration and multiplying it by 100, with the results being reported as medians and IQRs. By definition, the higher this percentage, the closer the DOAC concentrations were from peak to trough. The statistical analyses were performed by means of non-parametric tests (Mann–Whitney or Spearman’s rho), and *p* < 0.05 was considered as statistically significant. The data were analyzed by the SPSS statistical package (SPSS, Chicago, IL, USA).

## 5. Results

A total of 131 patients on DOACs [apixaban (*n* = 37), rivaroxaban (*n* = 34), dabigatran (*n* = 30), edoxaban (*n* = 30)] and 33 patients on VKAs were analyzed. The majority of the patients (80%) were on treatment for VTE, and the remaining 20% were treated because of atrial fibrillation. The VKA dosages were adjusted, aimed at an INR of 2.0–3.0. DOACs were prescribed at dosages consistent with the reasons for treatment and the patients’ characteristics (i.e., age, renal clearance, etc.). The patients’ age was comparable across the categories [median (min–max): VKAs 72 years (38–89); apixaban 70 (22–91); dabigatran 70 (43–88); edoxaban 65 (29–83)], except for rivaroxaban [median 55 (24–83)]. However, the linear regression analysis did not show any significant association between age and the thromboelastometry or thrombin generation parameters. Although the population of patients on DOAC was heterogenous, as DOAC dosages may differ for VTE and AF, and as DOAC dosages can influence the peak and trough levels, for the purpose of this study, we pooled the data before the analysis. This is reasonable as we aimed to evaluate the impact of plasma DOAC concentrations on global coagulation and to look for the correlation of plasma concentration and thromboelastometry or TGA, and these do not depend on the investigated population.

### 5.1. DOAC Concentrations

**Figure 1:**
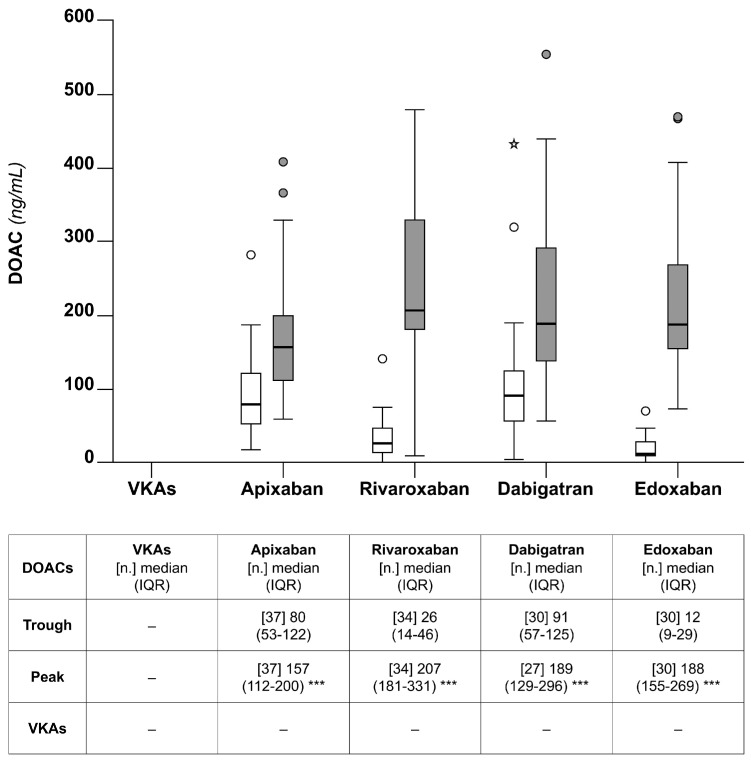
Box plots of the DOAC concentrations at the peak (grey) and the trough for the chronically anticoagulated patients included in this study. The table reports the numerical results. The *p*-values (* *p* < 0.05, ** *p* < 0.01, *** *p* < 0.001) refer to the non-parametric test for the paired data (Wilcoxon), trough vs. peak. The *p*-values (§ *p* < 0.05, §§ *p* < 0.01, §§§ *p* < 0.001) refer to the non-parametric test for the unpaired data (Mann–Whitney), trough or peak vs. VKAs.

Figure 1 shows the DOAC concentrations for the whole cohort at the peak and trough. As expected, the DOAC concentrations were higher at the peak than the trough. Furthermore, there was a relatively high interindividual variability for each of the four drugs. Apixaban showed the smallest interindividual variability at the peak.

The percentage of the DOAC concentration (IQR) measured at the trough relative to that measured at the peak that was obtained for apixaban was 49% (39–59). The corresponding values for the other DOACs were 54% (31–78) for dabigatran, 11% (8–21) for rivaroxaban, and 7% (4–11) for edoxaban.

### 5.2. Thromboelastometry

#### 5.2.1. Clotting Time (CT)

**Figure 2:**
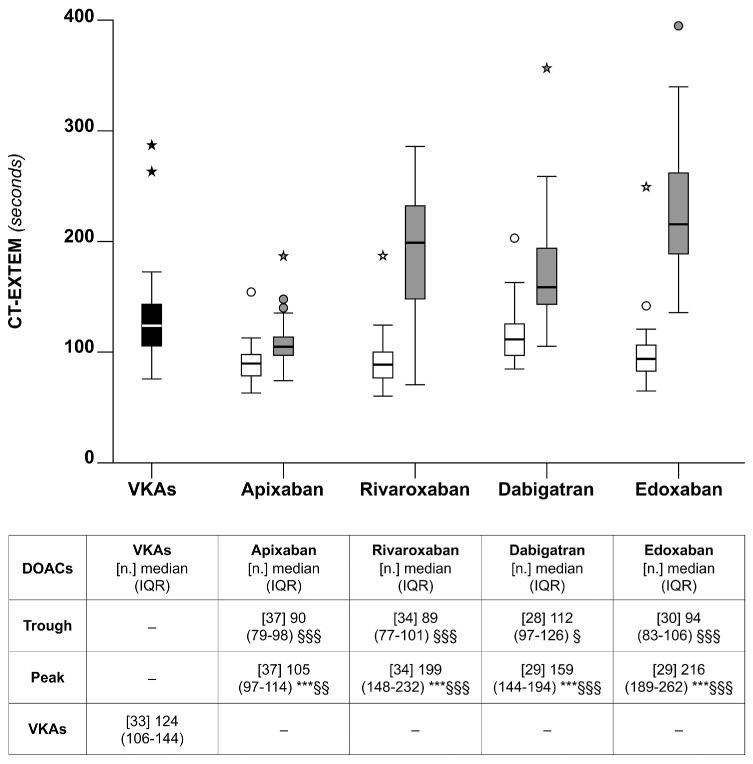
Box plots of the thromboelastometry parameters for clotting time (the CT-EXTEM), observed for the patients on chronic anticoagulation that were included in the study. See also the legends to Figure 1.

The median the CT-EXTEM was significantly lower for VKAs than for any of the DOACs at the peak (*p* < 0.01), except for apixaban (Figure 2). When the analysis was restricted to DOACs, the CT-EXTEM was the lowest for apixaban at the peak and the highest for edoxaban at the peak; the differences were marginal at the trough (Figure 2). The percentage of the CT-EXTEM measured at the trough relative to that measured at the peak that was obtained for apixaban was 83% (75–92). The corresponding values for the other DOACs were 70% (58–78) for dabigatran, 47% (41–56) for rivaroxaban, and 42% (36–49) for edoxaban. The pattern of variation for the CT-INTEM was substantially similar.

The correlation between the CT and the DOAC concentrations or the VKA-INR was highly significant (*p* < 0.001) (Table 1). Based on the interpolation of the data from the linear regression lines observed in this study (the CT vs. the DOAC concentrations), we calculated the CT values (95% CI) of 241 (233–249) or 89 (81–97) seconds, corresponding to DOAC concentrations of 400 or 20 ng/mL when all of the DOACs were included (Table 1). The corresponding the CT values for the individual DOACs were similar, except for apixaban, which showed a relatively narrow difference between the CT corresponding to 400 and that corresponding to 20 ng/mL (Table 1).

#### 5.2.2. Clot Formation Time (CFT)

**Figure 3:**
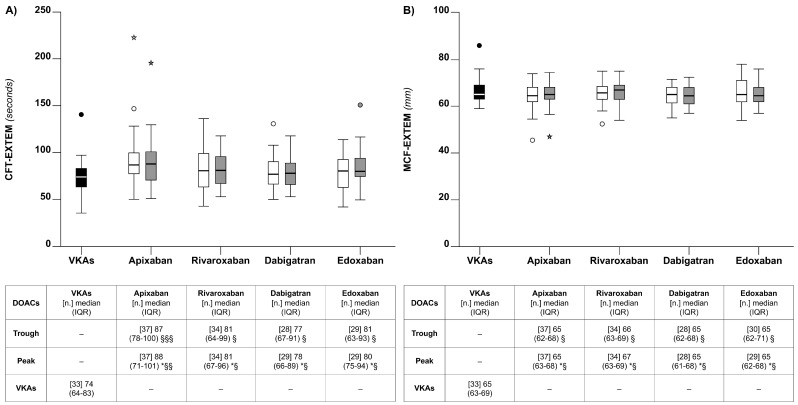
Box plots of the thromboelastometry parameters clot formation time (CFT) (**Panel A**) and maximal clot firmness (MCF) (**Panel B**), observed for the patients on chronic anticoagulation that were included in the study. See also the legends to Figure 1.

The median CFT-EXTEM was significantly lower for VKAs than for any of the DOACs at the peak or the trough (*p* < 0.05) (Figure 3). When the analysis was restricted to DOACs, CFT-EXTEM changes across the DOACs were marginal, both at the peak and the trough (Figure 3). The pattern of variation for CFT-INTEM was substantially similar.

There was no correlation between the CFT and the DOAC concentrations or the VKA-INR (*p* > 0.05) (Table 1).

#### 5.2.3. Maximal Clot Firmness (MCF)

There were relatively small differences between the median MCF-EXTEM for the VKAs and the DOACs and also between the DOACs (Figure 3). The pattern of variation for MCF-INTEM was substantially similar. There was no correlation between the MCF and the DOAC concentrations or the VKA-INR (*p* > 0.05) (Table 1).

### 5.3. Thrombin Generation

The patients on dabigatran were not evaluated for TGA as this drug in the test system inhibits the (internal) standard needed for results calculation.

#### 5.3.1. Lag Time

**Figure 4:**
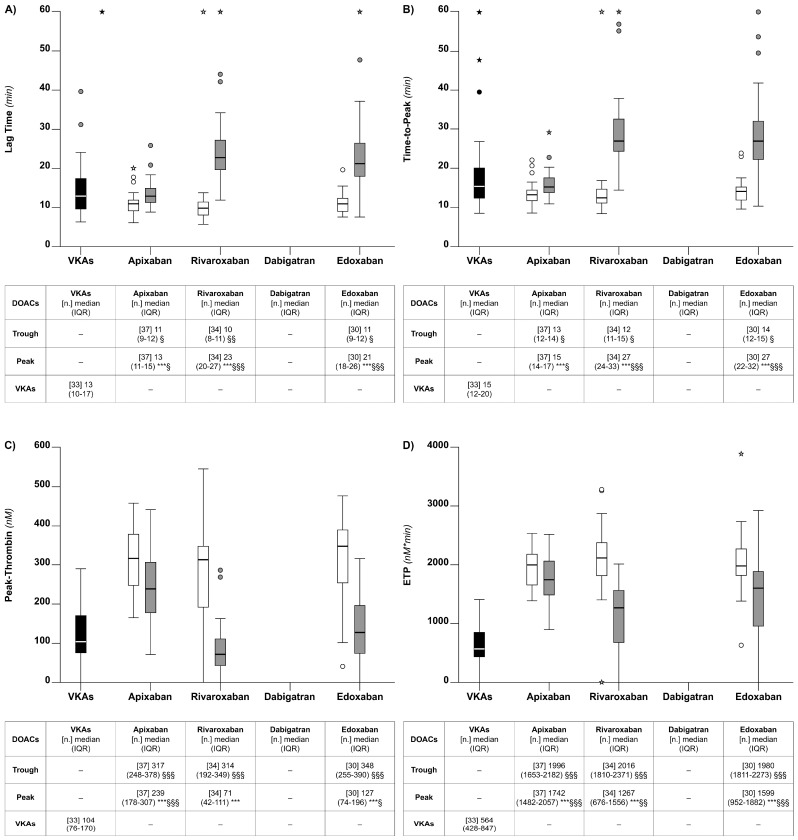
Box plots of the thrombin generation parameters observed for the patients on chronic anticoagulation that were included in the study. Lag time (**Panel A**). Time-to-peak (**Panel B**). Peak-thrombin (**Panel C**). Endogenous thrombin potential (ETP) (**Panel D**). See also legends to Figure 1.

The median lag time was significantly lower for VKAs than for any of the DOACs at the peak (*p* < 0.05), except for apixaban (Figure 4). When the analysis was restricted to the DOACs, the highest lag times were observed for rivaroxaban and edoxaban, and the lowest was observed for apixaban at the peak (Figure 4); there were relatively large differences (peak vs. trough), except for apixaban; the lag time differences were marginal across the DOACs at the trough (Figure 4). There was a high correlation between the lag time and the DOAC concentrations (rho = 0.70, *p* < 0.001); there was a trend for correlation between lag time and the VKA-INR that did not reach statistical significance (rho = 0.30, *p* = 0.08) (Table 2).

#### 5.3.2. Time-to-Peak

The median time-to-peak was significantly lower for VKAs than for the patients on DOACs at the peak (*p* < 0.001), except for apixaban (Figure 4); there were relatively large differences (peak vs. trough) for all of the DOACs, except for apixaban, but there were no obvious differences across the DOACs at the trough (Figure 4). While there was a strong correlation between the time-to-peak and the DOAC concentrations (rho = 0.66, *p* < 0.001) (Table 2), the correlation between the time-to-peak and the VKA-INR did not reach statistical significance (rho = 0.31, *p* = 0.08) (Table 2).

#### 5.3.3. Peak-Thrombin

The median peak-thrombin for the patients on VKAs was significantly lower than for any of the DOACs at the trough (*p* < 0.01) (Figure 4). Among the DOACs, the lowest peak-thrombin values were recorded for rivaroxaban and edoxaban at the peak (*p* < 0.001) (Figure 4). Relatively high median peak-thrombin levels were recorded for all of the DOACs at the trough, with marginal differences between the DOACs (Figure 4). There was a relatively large peak vs. trough difference for all of the DOACs, except for apixaban. While there was a strong correlation between peak-thrombin and the DOAC concentrations (rho = −0.67, *p* < 0.001) (Table 2), the correlation between peak-thrombin and the VKA-INR was weaker, though significant (rho = −0.53, *p* < 0.01) (Table 2).

#### 5.3.4. Endogenous Thrombin Potential (ETP)

The pattern of the ETP variation was similar to that of peak-thrombin. The Median ETP for the patients on VKAs was significantly lower than for any of the DOACs, regardless of whether it was measured at the peak or the trough (Figure 4). When the analysis was restricted to DOACs, the highest ETP median values were observed for apixaban and edoxaban at the peak (Figure 4). There were no clear differences across the DOACs when the ETP was measured at the trough (Figure 4). There was a relatively large difference for the peak vs. the trough for all of the DOACs, except for apixaban. While there was a strong correlation between the ETP and the DOAC concentrations (rho = −0.58, *p* < 0.001) (Table 2), the correlation between the ETP and the VKA-INR was weaker, though significant (rho = −0.51, *p* < 0.01) (Table 2). Based on the interpolation of the data from the linear regression line (the ETP vs. the DOAC concentrations) observed in this study, the ETP values (95% CI) of 914 (802–1026) or 2054 (1942–2166) nM x min, corresponding to DOAC concentrations of 400 or 20 ng/mL, were calculated when all of the DOACs were included. The corresponding values for the individual DOACs were similar, except for apixaban, which showed a relatively narrow difference between the ETP corresponding to 400 ng/mL and that corresponding to 20 ng/mL (Table 2). There were strong correlations between the thromboelastometry and TGA parameters (Table 3).

## 6. Discussion

While randomized trials have been exhaustive in showing the clinical characteristics of DOACs [1], less well defined are their dynamic characteristics in modifying global coagulation procedures, especially thrombin generation [7,8,9,10]. There are reports on thromboelastometry, especially for patients treated with some of the DOACs [11,12,13,14,15,16,17,18], but most of the studies were performed with homemade modifications of thromboelastometry, and little is known on how regular devices perform for patients on DOACs or VKAs. Furthermore, no face-to-face comparisons between VKAs and all of the DOACs have been reported with respect to thromboelastometry or thrombin generation. Finally, very little is known about the comparisons of global procedures for different DOACs when investigated at peak and trough plasma concentrations.

With this gap of knowledge, we undertook a comprehensive investigation that included the DOACs presently available and VKAs to assess for the variation induced by these drugs on thromboelastometry and thrombin generation. To our knowledge, this is the first comprehensive investigation of chronically anticoagulated patients, carried out simultaneously with the two global coagulation procedures. The results of the study may help to shed light on the dynamics of the thrombinogenesis induced by VKAs or DOACs and possibly to give information that is useful for patients’ management. The results of this study allow for the following considerations.

### 6.1. DOAC Concentrations

As expected [19], the plasma concentrations of DOACs were greater at the peak than trough (see Figure 1), indicating that the population included in this study is representative of the real-life population of anticoagulated patients. Interestingly, apixaban and dabigatran, at the trough, showed the highest median percentage concentrations relative to peak [i.e., apixaban, 49% (39–59); dabigatran, 54% (31–78)] than the other DOACs [i.e., rivaroxaban, 11% (8–21) and 7% edoxaban, (4–11)]. These results indicate that apixaban and dabigatran are characterized by relatively close median peak and trough levels. It cannot be excluded that these results may (at least in part) depend on the twice-daily regimen used for these drugs. However, in contrast to the similar median percentage concentration from peak to trough, dabigatran showed a larger interindividual variability [i.e., IQR dabigatran (31–78)] than apixaban [i.e., IQR apixaban, (39–59)]; this difference is statistically significant (*p* < 0.05). Hence, it is unlikely that the twice-daily regimen can be taken as an explanation for the above results. Cumulatively, these results indicate that apixaban, when assessed for the whole population of chronically anticoagulated patients, promotes better stability of plasma concentrations over the daily course of treatment than the other DOACs.

### 6.2. Thromboelastometry

VKAs were, in general, less effective than DOACs at inducing anticoagulation when assessed with the CT (see Figure 2). When the analysis was restricted to DOACs, apixaban and edoxaban, at the peak, showed the smallest and the greatest CT prolongation, respectively (see Figure 2). The usefulness of the CT to evaluate the anticoagulation achieved by DOACs, as shown in our study, confirms previous observations in patients receiving some of the DOACs [13] and extends these observations to all the DOACs that are currently available. The CT was the only thromboelastometry parameter that was strongly correlated with DOAC concentrations or the INR for patients on VKAs. In contrast, the other parameters, CFT and MCF, were not correlated (see Table 1). Hence, CFT and MCF were unable to show the anticoagulation induced by VKAs or DOACs. The different behavior of thromboelastometry parameters in response to anticoagulation has already been reported by other authors. The reasons are, however, unknown. One may plausibly argue that anticoagulant drugs affect much more the initial velocity of clot formation (identified by the CT) than the subsequent velocity of clot formation and the clot firmness (identified as CFT and MCF, respectively). Although the absolute value to be given to the CT prolongation in patients taking DOACs cannot be accurately determined, we roughly estimated the CT values corresponding to DOAC concentrations of 400 or 20 ng/mL that may be considered as over- or under-dosages (see Table 1). Since thromboelastometry devices are often available in emergency departments, the measurement of the CT might be useful for the urgent management of bleeding patients when dedicated tests for DOAC concentrations are not available.

### 6.3. Thrombin Generation

The results indicate that both VKAs and DOACs were, in general, able to alter the TGA parameters, but to different extents. For example, the parameters related to the velocity of thrombin generation (i.e., lag time and time-to-peak) were, on average, similarly altered by VKAs or DOACs (see Figure 4). In contrast, the parameters related to the quantity of thrombin generation (i.e., peak-thrombin and the ETP) were cumulatively more altered by VKAs than DOACs (see Figure 4). Whether the above differences are related to the efficacy/safety of the two types of drugs is unknown. When the analysis was restricted to DOACs, the results showed that the TGA parameters for rivaroxaban or edoxaban were more effective at showing the levels of anticoagulation than apixaban. Notably, these results contrast with those of the thromboelastometry parameters (see above), whereby the time needed for clot formation (i.e., CT) seems to reflect the levels of anticoagulation achieved by DOACs better than either the velocity of clot formation (i.e., CFT) or the stability or strength of clots (i.e., MCF).

Based on the strong correlation between the ETP and DOAC concentrations, we roughly estimated that the critical value of the ETP corresponds to the DOAC concentration of 400 or 20 ng/mL (see Table 2). These critical values could be taken as a guide to make decisions on the level of anticoagulation in an emergency, whenever DOAC concentration measurements are not available. It should, however, be recognized that the practical value of this information is relatively little, as TGA is not presently available in most clinical laboratories.

### 6.4. Anticoagulation at Peak and Trough

As expected, patients on DOACs, at the peak, presented with a relatively greater degree of anticoagulation than at trough when assessed by thromboelastometry or TGA. Based on the following considerations, we propose that the above observations may have pathophysiological implications. The thromboelastometry parameter CT and the TGA parameter ETP are strongly correlated with DOAC concentrations. On the other hand, DOAC concentrations are considered as reliable risk factors for recurrent thrombosis or hemorrhage when they are low or high, respectively [20,21]. Hence, if one takes the DOAC concentrations as reliable indexes of anticoagulation being achieved during treatment, also the CT and the ETP should be considered similarly. Consequently, it is tempting to speculate that the results of the present study would suggest that patients taking some of the DOACs are not equally anticoagulated during the course of daily treatment when their plasma concentrations are at the peak or trough, and there are no studies assessing whether thrombosis recurrences are more likely to occur at the trough than at peak. In this respect, apixaban is a case of interest. This drug, when compared to other DOACs, showed, in fact, a relatively modest difference at the peak-vs-trough for all the parameters measured in our study [i.e., drug concentrations (see Figure 1), the CT (see Figure 2), and the ETP (see Figure 4)]. The above results would favor the hypothesis that apixaban ensures a more stable anticoagulation over the daily course of treatment than do the other DOACs.

### 6.5. Pathophysiological Considerations

Cumulatively, the above results might have pathophysiological relevance concerning the safety/efficacy and mechanism of action of different DOACs. A recent population-based cohort study concluded that VTE patients, who were new users of apixaban, had lower rates of bleeding or recurrent VTE than new users of rivaroxaban [22]. Another study, based on a network metanalysis for patients with AF, reported that apixaban was associated with a significantly lower risk of major bleeding than other DOACs, and dabigatran was associated with a significantly lower risk of major bleeding than rivaroxaban and warfarin [23]. The above conclusions are in line with the results of our study. As a matter of fact, the patients on apixaban in our study presented with a smaller degree of anticoagulation, as assessed by thromboelastometry (a relatively low CT) or thrombin generation (relatively high peak-thrombin or ETP), than those on rivaroxaban or other DOACs (see Figure 2 and Figure 4). In addition, apixaban, in our study, promoted a better stability of plasma concentration over the daily course of treatment compared to the other DOACs (see Figure 1). The better stability of plasma concentration shown by apixaban corresponds to a more stable anticoagulation from the peak to trough. Apixaban showed, in fact, a relatively high median percentage of the CT from the peak to trough [i.e., 83% (75–92)] when compared to the other DOACs. This could justify the lower rates of adverse events observed for apixaban when compared to the other DOACs [22,23]. Finally, it cannot be excluded that apixaban mediates its anticoagulant properties through other, yet unknown, mechanisms that operate outside coagulation. Indeed, apixaban has been reported to affect platelet activity when investigated under flow conditions [24].

### 6.6. Strengths and Limitations

The strengths of this study are the simultaneous and comprehensive investigation of two large groups of patients being treated with VKAs or DOACs by means of the most important global coagulation procedures that are currently available. This allowed us to derive important pathophysiological considerations on the mechanisms of the action of VKAs vs. DOACs, as well as considerations that may be useful for patients’ management.

Among the limitations, we should mention the cross-sectional nature of the study, which did not allow us to draw conclusions on the association of the thromboelastometry or TGA results with clinical events. However, since the parameters of thromboelastometry and TGA are strongly correlated with DOAC concentrations, there is no rationale to expect that thromboelastometry and TGA are not associated with the risk of adverse events in patients who present with extreme values of the parameters measured by the global procedures. TGA was performed using relatively low tissue factor concentrations (i.e., 1 pM). The concentration of the tissue factor is known to affect TGA. However, since the aim of the study was the face-to-face comparison of different anticoagulant drugs, it is unlikely that higher concentrations of the tissue factor would change the results and conclusions. We did not assess dabigatran for TGA as this drug inhibits the thrombin standard used for testing. The addition of idarucizumab (a dabigatran neutralizer) into the test system has been used in other reports [25] to make TGA feasible for patients on dabigatran. We did not undertake this modification because of the cost. Finally, we restricted our study to patients who were mainly being treated for VTE or AF. There may be other settings where anticoagulation with DOACs is used that would require the same sort of investigation.

### 6.7. Conclusions

We investigated herein relatively large numbers of well-characterized patients on chronic anticoagulation and showed that thromboelastometry and TGA are suited to assess the degree of anticoagulation achieved by VKAs or DOACs. Overall, the degree of anticoagulation achieved by both types of drugs is similar, regardless of whether it is evaluated by thromboelastometry or TGA. There are, however, important differences related to the specific parameters. While the thromboelastometry parameter, defined as the CT, is responsive to the anticoagulation induced by both types of drugs, the other parameters (CFT or MCF) are not. On the other hand, the anticoagulation achieved by both types of drugs was detected by the TGA parameters, but to different extents. The parameters related to the velocity of thrombin generation (lag time and time-to-peak) were less modified by both types of drugs than the parameters related to the quantity of thrombin generation (peak-thrombin or ETP). Based on the correlation between the CT or the ETP and DOAC concentrations, we estimated the critical values of the two parameters that correspond to DOAC concentrations of 400 or 20 ng/mL, which can be approximately taken as values defining over- or under-dosages. This information could be taken as a guide to manage patients with thrombosis or hemorrhage who are referred to emergency departments where thromboelastometry devices are often available, but dedicated assays for DOAC concentration measurements are not.

## Figures and Tables

**Table 1:** Correlations of the thromboelastometry parameters vs. the DOAC concentrations or the VKA-INR, as measured for patients on chronic anticoagulation. Peak + trough DOAC concentrations were included in this analysis. CT-EXTEM or INTEM, coagulation time determined with extrinsic or intrinsic reagents. CFT-EXTEM or INTEM, clot formation time. MCF-EXTEM or INTEM, maximal clot firmness. VKA-INR, international normalized ratio for patients on VKAs. * Based on the linear regression line (DOAC concentration vs. the thromboelastometry CT).

Thromboelastometry Parameters vs. DOAC Concentrations			CT Value (95% CI) Corresponding to DOAC Concentrations of *	
CT-EXTEM (seconds)	Rho	*p* value	400 ng/mL	20 ng/mL
DOACs (all)	0.725	<0.001	241 (233–249)	89 (81–97)
Apixaban	0.667	<0.001	150 (144–156)	78 (72–84)
Rivaroxaban	0.788	<0.001	236 (222–250)	99 (85–113)
Dabigatran	0.824	<0.001	224 (212–236)	91 (79–103)
Edoxaban	0.86	<0.001	341 (331–351)	105 (95–115)
**CFT-EXTEM** (seconds)				
DOAC (all)	0.010	0.87		
Apixaban	0.065	0.58		
Rivaroxaban	−0.018	0.88		
Dabigatran	0.047	0.73		
Edoxaban	0.053	0.69		
**MCF-EXTEM** (mm)				
DOAC (all)	0.013	0.87		
Apixaban	−0.044	0.71		
Rivaroxaban	0.021	0.86		
Dabigatran	0.213	0.12		
Edoxaban	0.040	0.76		
**CT-INTEM** (seconds)				
DOAC (all)	0.49	<0.001		
Apixaban	0.23	<0.05		
Rivaroxaban	0.62	<0.001		
Dabigatran	0.75	<0.001		
Edoxaban	0.53	<0.001		
**CFT-INTEM** (seconds)				
DOAC (all)	0.066	0.29		
Apixaban	0.23	<0.05		
Rivaroxaban	0.12	0.35		
Dabigatran	−0.04	0.57		
Edoxaban	0.001	0.99		
**MCF-INTEM** (mm)				
DOAC (all)	0.08	0.21		
Apixaban	−0.08	0.46		
Rivaroxaban	0.08	0.51		
Dabigatran	0.33	<0.05		
Edoxaban	0.08	0.57		
**Thromboelastometry parameters vs. the VKA-INR**				
CT-EXTEM	0.58	<0.001		
CFT-EXTEM	0.07	0.71		
MCF-EXTEM	−0.33	0.06		
CT-INTEM	0.45	<0.01		
CFT-INTEM	0.32	0.07		
MCF-INTEM	−0.16	0.38		

**Table 2:** Correlations of thrombin generation parameters vs. DOAC concentrations or the VKA-INR as measured for patients on chronic anticoagulation. Peak + trough DOAC concentrations were included in this analysis. the VKA-INR, international normalized ratio measured for patients on VKAs. * Based on the linear regression line (DOAC concentration vs. thrombin potential, ETP).

Thrombin Generation Assay Parameters vs. DOAC Concentrations			ETP Value (95% CI) Corresponding to DOAC Concentration of *	
Lag time (minutes)	Rho	*p* value	400 ng/mL	20 ng/mL
DOACs (all)	0.70	<0.001		
Apixaban	0.48	<0.001		
Rivaroxaban	0.69	<0.001		
Edoxaban	0.82	<0.001		
**Time-to-peak** (minutes)				
DOAC (all)	0.66	<0.001		
Apixaban	0.47	<0.001		
Rivaroxaban	0.71	<0.001		
Edoxaban	0.83	<0.001		
**Peak-thrombin** (nM)				
DOAC (all)	−0.67	<0.001		
Apixaban	−0.63	<0.001		
Rivaroxaban	−0.73	<0.001		
Edoxaban	−0.76	<0.001		
**Endogenous thrombin potential** (nMxmin)				
DOAC (all)	−0.58	<0.001	914 (802–1026)	2054 (1492–2166)
Apixaban	−0.47	<0.001	1278 (1140–1416)	2038 (1.900–2176)
Rivaroxaban	−0.65	<0.001	473 (250–696)	1993 (1770–2216)
Edoxaban	−0.57	<0.001	867 (662–1072)	2007 (1802–2212)
**Thrombin generation assay parameters vs. the VKA-INR**				
Lag time	0.30	0.08		
Time-to-peak	0.31	0.08		
Peak-thrombin	−0.53	<0.01		
Endogenous thrombin potential	−0.51	<0.01		

**Table 3:** Correlations of thrombin generation assay vs. thromboelastometry parameters for patients on DOACs or VKAs. DOAC peak + trough values were included in this analysis. CT-EXTEM, coagulation time determined with extrinsic reagents.

Patients on DOACs		
Lag time vs. CT-EXTEM	Rho	*p* value
DOAC (all)	0.81	<0.001
Apixaban	0.57	<0.001
Rivaroxaban	0.87	<0.001
Edoxaban	0.83	<0.001
**Time-to-peak vs. CT-EXTEM**		
DOAC (all)	0.82	<0.001
Apixaban	0.55	<0.001
Rivaroxaban	0.86	<0.001
Edoxaban	0.83	<0.001
**Peak-thrombin vs. CT-EXTEM**		
DOAC (all)	−0.67	<0.001
Apixaban	−0.37	<0.001
Rivaroxaban	−0.82	<0.001
Edoxaban	−0.65	<0.001
**Endogenous thrombin potential vs. CT-EXTEM**		
DOAC (all)	−0.52	<0.001
Apixaban	−0.20	0.09
Rivaroxaban	−0.72	<0.001
Edoxaban	−0.51	<0.001
**Patients on VKAs**		
Lag time vs. CT-EXTEM	0.72	<0.001
Time-to-peak vs. CT-EXTEM	0.72	<0.001
Peak-thrombin vs. CT-EXTEM	−0.79	<0.001
Endogenous thrombin potential vs. CT-EXTEM	−0.75	<0.001

## Data Availability

The original contributions presented in the study are included in the article, further inquiries can be directed to the corresponding author.
